# A new smoothing modified three-term conjugate gradient method for $l_{1}$-norm minimization problem

**DOI:** 10.1186/s13660-018-1696-9

**Published:** 2018-05-03

**Authors:** Shouqiang Du, Miao Chen

**Affiliations:** School of Mathematics and Stochastic, Qingdao University, Qingdao, China

**Keywords:** 90C30, 90C33, Nonsmooth optimization problem, Smoothing modified three-term conjugate gradient method, Global convergence

## Abstract

We consider a kind of nonsmooth optimization problems with $l_{1}$-norm minimization, which has many applications in compressed sensing, signal reconstruction, and the related engineering problems. Using smoothing approximate techniques, this kind of nonsmooth optimization problem can be transformed into a general unconstrained optimization problem, which can be solved by the proposed smoothing modified three-term conjugate gradient method. The smoothing modified three-term conjugate gradient method is based on Polak–Ribière–Polyak conjugate gradient method. For the Polak–Ribière–Polyak conjugate gradient method has good numerical properties, the proposed method possesses the sufficient descent property without any line searches, and it is also proved to be globally convergent. Finally, the numerical experiments show the efficiency of the proposed method.

## Introduction

In this paper, we consider the following nonsmooth optimization problems with $l_{1}$-norm minimization problem

1 $$ \min_{x \in R^{n}}\frac{1}{2}\|Ax - b\|_{2}^{2} + \tau \|x\|_{1}, $$

where $x \in R^{n}$, $A \in R^{m \times n}$ ($m \ll n$), $b \in R^{m}$, $\tau> 0$, $\Vert v \Vert _{2}$ denotes the Euclidean norm of *v* and $\|v\|_{1} = \sum_{i = 1}^{n} |v_{i}|$ is the $l_{1}$-norm of *v*. This problem is widely used in compressed sensing, signal reconstruction, analog-to-information conversion and related to many mathematical problems [1–16]. Problem (1) is also a typical compressed sensing scenario, which can reconstruct a length-*n* sparse signal from *m* observations. From the Bayesian perspective, problem (1) can also be seen as a maximum a posteriori criterion for estimating *x* from observations $b = Ax + \omega$, where *ω* is the Gaussian noise of variance $\sigma^{2}$. Many researchers have studied the numerical algorithms, which can be used to solve problem (1) with large-scale data such as fixed point method [1], gradient projection method for sparse reconstruction [2], interior-point continuation method [3, 4], iterative shrinkage thresholds algorithms in [5, 6], linearized Bregman method [7, 8], alternating direction algorithms [9], nonsmooth equations-based method [10] and some related methods [11, 12]. Just recently, a smoothing gradient method has been given for solving problem (1) based on the new transformed absolute value equations in [14, 15]. The transformation is based on the equivalence between a linear complementarity problem and an absolute value equation problem [17, 18]. The complementarity problem, the absolute value equation problem, and the related constrained optimization problem are three kinds of important optimization problems [19–23]. On the other hand, the nonlinear conjugate gradient methods and smoothing methods are used widely to solve large-scale optimization problems [24, 25], total variation image restoration [26], monotone nonlinear equations with convex constraints [27], and nonsmooth optimization problems, such as nonsmooth nonconvex problems [28], minimax problem [29], P0 nonlinear complementarity problems [30]. Specially, the effectiveness of widely used and attained different numerical outcomes three-term conjugate gradient method, which is based on Hang–Zhang conjugate gradient method and Polak–Ribière–Polyak conjugate gradient method [31–33], has been widely studied. Based on the above analysis, in this paper, we propose a new smoothing modified three-term conjugate gradient method to solve problem (1). The global convergence analysis of the proposed method is also presented.

The remainder of this paper is organized as follows. In Sect. 2, we give the transformation of problem (1), which includes the transformation of a linear complementarity problem transformed into an absolute value equation problem. In Sect. 3, we present the smoothing modified three-term conjugate gradient method and give the convergence analysis of it. Finally, we give some numerical results of the given method which show the effectiveness of it.

## Results: the transformation of the problem

In this section, as in [9, 10, 14, 15], we set

$${x} = {u} - {v},\quad{u}\geq0,{v}\geq0, $$

where ${u}_{{i}} = ({x}_{{i}})_{ +} $ and ${v}_{{i}} = ( - {x}_{{i}})_{ +} $ for all ${i} = 1,2, \ldots, {n}$ with $({x}_{{i}})_{ +} = \max\{ x_{i},0\}$. And we also have $\Vert {x} \Vert _{1} = 1_{n}^{T}u + 1_{n}^{T}v$, where $1_{n} = [1,1, \ldots,1]^{T}$ is an *n*-dimensional vector with n ones. Thus, problem (1) can be transformed into the following problem:

$$\min_{z = (u,v)^{T} \ge0}\frac{1}{2}\|b - Az\|_{2}^{2} + \tau 1_{n}^{T}u + \tau1_{n}^{T}v, $$

i.e.,

2 $$ \min_{z \ge0}\frac{1}{2}z^{T}Hz + c^{T}z, $$

where

$$z = (u,v)^{T},\qquad c = \tau1_{2n} + \left ( \textstyle\begin{array}{c} - {c} ^{ -} \\ {c} ^{ -} \end{array}\displaystyle \right ),\qquad c = A^{T}b,\qquad H = \left ( \textstyle\begin{array}{c@{\quad}c} A^{T}A & - A^{T}A \\ - A^{T}A & A^{T}A \end{array}\displaystyle \right ). $$

Since *H* is a positive semi-definite matrix, problem (2) can be translated into a linear variable inequality problem, which is to find $z \in R^{2n}$ such that

3 $$ \langle Hz + c,\tilde{z} - z \rangle\ge0, \quad\forall\tilde{z} \ge0. $$

By the feasible structure of the feasible region of *z*, problem (3) is equivalent to the linear complementary problem, which to find $z \in R^{2n}$ such that

4 $$ z \ge0,\qquad Hz + c \ge0,\qquad z^{T}(Hz + c) = 0. $$

Due to the equivalence of linear complementarity problems and absolute value equation problems, problem (4) can be transformed into the following absolute value equation problem, which is defined by

$$(H + I)z + c = \big|(H - I)z + c\big|. $$

Then problem (1) can be transformed into the following problem:

5 $$ \min_{z \in R^{2n}}f(z) = \frac{1}{2}\big\| (H + I)z + c - \big|(H - I)z + c\big|\big\| ^{2}. $$

## Main results and discussions

In this section, we present the smoothing modified three-term conjugate gradient method to solve problem (1). Firstly, we give the definition of smoothing function and smoothing approximation function of the absolute value function [14, 15, 29].

### Definition 1

Let $f:R^{n} \to R$ be a local Lipschitz continuous function. We call $\tilde{f}:R^{n} \times R_{ +} \to R$ a smoothing function of *f*, if

$$\lim_{\mu\to0}\tilde{f} (x) = f(x), $$

where $f_{\mu} ( \cdot)$ is continuously differentiable in $R^{n}$ for any fixed $\mu> 0$.

The smoothing function of the absolute value function is defined by

6 $$ \Phi_{i\mu} (z) = \sqrt{\bigl((H - I)z + c\bigr)_{i}^{2} + \mu^{2}} ,\quad\mu\in R_{ +} , i = 1,2, \ldots,2n, $$

and satisfies

$$\lim_{\mu\to0}\Phi_{i\mu} (z) = \big|\bigl((H - I)z + c \bigr)_{i}\big|,\quad i = 1,2, \ldots,2n. $$

Based on (6), we obtain the following unconstrained optimization problem:

$$\min_{z \in R^{2n}}\bar{f}_{\mu} (z) = \frac{1}{2}\sum _{i = 1}^{2n} \bar{f}_{i\mu}^{2} (z), $$

where $\bar{f}_{i\mu}(z) = ((H + I)z + c)_{i} - \Phi_{i\mu} (z)$ is a smoothing function of $f(z)$ in (5) for $i = 1,2, \ldots,2n$.

Now, we give the smoothing modified three-term conjugate gradient method.

### Algorithm 1

(Smoothing modified three-term conjugate gradient method)

Step 0. Choose $0 < \sigma< 1$, $0 < \rho< 1$, $r > 0$, $\mu= 2$, $\eta= 1$, $\varepsilon> 0$, $\mu_{0} > 1$ and, given an initial point $z_{0} \in R^{n}$, let $d_{0} = - \tilde{g}_{0} $, where $\tilde{g}_{0} = \nabla_{z}\tilde{f} (z_{0},\mu_{0})$.
Step 1. If $\|\nabla_{{z}}\tilde{f} \| \le \varepsilon$, stop; otherwise, go to Step 2.
Step 2. Compute search direction by using $\tilde{\beta}_{k}^{\mathrm {BZAU}}$ and $\tilde{\theta}_{k}^{\mathrm{BZAU}}$, which are defined by
  7 $$\begin{aligned}& \tilde{\beta}_{k}^{\mathrm{BZAU}} = \frac{\nabla_{Z}\tilde{f}_{\mu} (z_{k})^{T}(\nabla_{Z}\tilde{f}_{\mu} (z_{k}) - \nabla_{Z}\tilde{f}_{\mu} (z_{k - 1}))}{ - \eta \nabla_{Z}\tilde{f}_{\mu} (z_{k - 1})^{T}d_{k - 1} + \mu |\nabla_{Z}\tilde{f}_{\mu} (z_{k})^{T}d_{k - 1}|}, \end{aligned}$$
  8 $$\begin{aligned}& \tilde{\theta}_{k}^{\mathrm{BZAU}} = \frac{\nabla_{Z}\tilde{f}_{\mu} (z_{k})^{T}d_{k - 1}}{ - \eta\nabla_{Z}\tilde{f}_{\mu} (z_{k - 1})^{T}d_{k - 1} + \mu|\nabla_{Z}\tilde{f}_{\mu} (z_{k})^{T}d_{k - 1}|}, \\& d_{k} = \left \{ \textstyle\begin{array}{l@{\quad}l} - \nabla_{Z}\tilde{f}_{\mu} (z_{k}) & \text{if } k = 0, \\ - \nabla_{Z}\tilde{f}_{\mu} (z_{k}) + \tilde{\beta}_{k}^{\mathrm{BZAU}}d_{k - 1} - \tilde{\theta}_{k}^{\mathrm {BZAU}}y_{k - 1} & \text{if } k \ge1, \end{array}\displaystyle \right . \end{aligned}$$
  where $y_{k - 1} = \nabla_{Z}\tilde{f}_{\mu} (z_{k}) - \nabla_{Z}\tilde{f}_{\mu} (z_{k - 1})$.
Step 3. Compute $\alpha_{k}$ by the Armijo line search, where $\alpha _{k} = \max \{ \rho^{0},\rho^{1},\rho^{2}, \ldots \}$ and $\rho^{i}$ satisfies
  9 $$ \tilde{f} \bigl(z_{k} + \rho^{i}d_{k}, \mu_{k}\bigr) \le \tilde{f} (z_{k},\mu_{k}) + \sigma \rho^{i}\nabla_{Z}\tilde{f}_{\mu} (z_{k})^{T}d_{k}. $$
Step 4. Compute $z_{k + 1} = z_{k} + \alpha_{k}d_{k}$, if $\|\nabla_{z}\bar{f}(z_{k + 1},\mu_{k})\| \ge r\mu_{k}$, set $\mu_{k + 1} = \mu_{k}$. Otherwise, let $\mu_{k + 1} = \sigma \mu_{k}$.
Step 5. Set $k: = k + 1$ and go to Step 1.

Now, we give convergence analysis of Algorithm 1. In order to get the global convergence of Algorithm 1, we give the following assumptions.

### Assumption 1

(i) The level set $\Omega= \{ z \in R^{2n}|\tilde{f}_{\mu} (z) \le \tilde{f}_{\mu} (z_{0}) \} $ is bounded.
(ii) There exists a positive constant $L > 0$ such that $\nabla_{Z}\tilde{f}_{\mu} (z_{k})$ is Lipschitz continuous on an open convex set $B \subseteq\Omega$ and for any $z_{1},z_{2} \in B$, i.e.,
  $$\big\| \nabla_{Z}\tilde{f}_{\mu} (z_{1}) - \nabla_{Z}\tilde{f}_{\mu} (z_{2})\big\| \le L \big\| z_{1} - z_{2}\big\| . $$
(iii) There exists a positive constant *m* such that
  $$m \Vert d_{x} \Vert ^{2} \le d^{T} \nabla_{z}^{2}\tilde{f}_{\mu} (z_{k})d_{k}, \quad\forall x,d \in R^{n}, $$
  where $\nabla_{z}^{2}\tilde{f}_{\mu} (z_{k})$ is the Hessian matrix of *f̃*.

By Assumption 1, we can see that there exist positive constants $\gamma> 0$ and *b* such that

$$\big\| \nabla_{Z}\tilde{f}_{\mu} (z_{k})\big\| \le\gamma,\quad \forall z_{k} \in \Omega $$

and

$$\|z_{1} - z_{2}\| \le b,\quad\forall z_{1},z_{2} \in \Omega. $$

### Lemma 1

*Suppose* $\{ {z}_{{k}} \}$ *and* $\{ {d}_{{k}} \}$ *are generated by Algorithm *1, *then*

$$\nabla_{Z}\tilde{f}_{\mu} (z_{k})^{T}d_{k} = - \bigl\Vert \nabla_{Z}\tilde{f}_{\mu} (z_{k}) \bigr\Vert ^{2} $$

*and*

$$\bigl\Vert \nabla_{Z}\tilde{f}_{\mu} (z_{k}) \bigr\Vert \le \Vert d_{k} \Vert . $$

### Proof

By Algorithm 1, we have

$$d_{k} = - \nabla_{Z}\tilde{f}_{\mu} (z_{k}) + \tilde{\beta}_{k}^{\mathrm{BZAU}}d_{k - 1} - \tilde{\theta}_{k}^{\mathrm {BZAU}}y_{k - 1}. $$

Multiplying both sides of the above equation by $\nabla_{Z}\tilde{f}_{\mu} (z_{k})^{T}$, we obtain

$$\begin{aligned} \nabla_{Z}\tilde{f}_{\mu} (z_{k})^{T}d_{k} ={}& {-} \big\| \nabla_{Z} \tilde{f}_{\mu} (z_{k})\big\| ^{2} + \frac{\nabla_{Z}\tilde{f}_{\mu} (z_{k})^{T}(\nabla_{Z}\tilde{f}_{\mu} (z_{k}) - \nabla_{Z}\tilde{f}_{\mu} (z_{k - 1}))(\nabla_{Z}\tilde{f}_{\mu} (z_{k})^{T}d_{k - 1})}{ - \eta\nabla_{Z}\tilde{f}_{\mu} (z_{k - 1})^{T}d_{k - 1} + \mu|\nabla_{Z}\tilde{f}_{\mu} (z_{k})^{T}d_{k - 1}|} \\ &- \frac{(\nabla_{Z}\tilde{f}_{\mu} (z_{k})^{T}d_{k - 1}\nabla_{Z}\tilde{f}_{\mu} (z_{k})^{T}(\nabla_{Z}\tilde{f}_{\mu} (z_{k}) - \nabla_{Z}\tilde{f}_{\mu} (z_{k - 1}))}{ - \eta \nabla_{Z}\tilde{f}_{\mu} (z_{k - 1})^{T}d_{k - 1} + \mu |\nabla_{Z}\tilde{f}_{\mu} (z_{k})^{T}d_{k - 1}|} ,\end{aligned} $$

i.e.,

$$\nabla_{Z}\tilde{f}_{\mu} (z_{k})^{T}d_{k} = - \big\| \nabla_{Z}\tilde{f}_{\mu} (z_{k}) \big\| ^{2}. $$

Now, we have

$$\big|\nabla_{Z}\tilde{f}_{\mu} (z_{k})^{T}d_{k}\big| = \big\| \nabla_{Z}\tilde{f}_{\mu} (z_{k}) \big\| ^{2} $$

and

$$\big|\nabla_{Z}\tilde{f}_{\mu} (z_{k})^{T}d_{k}\big| \le \big\| \nabla_{Z}\tilde{f}_{\mu} (z_{k})\big\| \Vert d_{k} \Vert . $$

By

$$\big\| \nabla_{Z}\tilde{f}_{\mu} (z_{k}) \big\| ^{2} \le \big\| \nabla_{Z}\tilde{f}_{\mu} (z_{k})\big\| \Vert d_{k} \Vert $$

we have

$$\big\| \nabla_{Z}\tilde{f}_{\mu} (z_{k})\big\| \le \Vert d_{k} \Vert . $$

Hence, the proof is complete. □

### Lemma 2

*Suppose Assumption* 1 *holds and* $\{ {z}_{{k}} \}$ *and* $\{ {d}_{{k}} \}$ *are generated by Algorithm *1, *then*

$$\sum_{k = 0}^{\infty} \frac{(\nabla_{Z}\tilde{f}_{\mu} (z_{k})^{T}d_{k})^{2}}{\|d_{k}\|^{2}} < + \infty $$

*and*

$$\sum_{k = 0}^{\infty} \frac{\|\nabla_{Z}\tilde{f}_{\mu} (z_{k})\|^{4}}{\|d_{k}\|^{2}} < + \infty. $$

### Proof

Using the techniques similar to lemmas in [31–33], we can get this lemma. The description will not be repeated again. □

### Lemma 3

*Suppose Assumption* 1 *holds and* $x_{k}$ *and* $d_{k}$ *are generated by Algorithm *1, *then*

10 $$ a_{1}\alpha_{k}\|d_{k}\|^{2} \le- \nabla_{Z}\tilde{f}_{\mu} (z_{k})^{T}d_{k}, $$

*where* $a_{1} = (1 - \sigma)^{ - 1}(m/2)$, *m* *is a positive constant and* $0 < \sigma< 1$.

### Proof

By using Taylor’s expansion, we have

11 $$ \tilde{f} (z_{k + 1}) = \tilde{f} (z_{k}) + \nabla_{Z}\tilde{f}_{\mu} (z_{k})^{T}s_{k} + \frac{1}{2}s_{k}^{T}G_{k}s_{k}, $$

where $s_{k} = z_{k + 1} - z_{k} = \alpha_{k}d_{k}$ and

$$G_{k} = \int_{0}^{1} \nabla_{z}^{2} \tilde{f}_{\mu} (z_{k} + \tau s_{k})\,d\tau s_{k}. $$

By Armijo line search, we know that

12 $$ \tilde{f} (z_{k + 1}) \le\tilde{f} (z_{k}) + \sigma \nabla_{z}\tilde{f}_{\mu} (z_{k})^{T}s_{k}. $$

By (11) and (12), we have

$$\frac{1}{2}s_{k}^{T}G_{k}s_{k} \le(1 - \sigma) \bigl( - \nabla_{Z}\tilde{f}_{\mu} (z_{k})^{T}s_{k}\bigr), $$

i.e.,

$$\frac{1}{2}(1 - \sigma)^{ - 1}m\alpha_{k} \|d_{k}\|^{2} \le- \nabla_{Z}\tilde{f}_{\mu} (z_{k})^{T}d_{k}. $$

Denote $a_{1} = (1 - \sigma)^{ - 1}(m/2)$, we get (10). Thus, we complete the proof. □

By Lemmas 1, 2, and 3, we can get global convergence of the given method, i.e., the following theorem.

### Theorem 1

*Suppose Assumption* 1 *holds*, *then*

$$\lim_{k \to\infty} \big\| \nabla_{Z}\tilde{f}_{\mu} (z_{k})\big\| = 0. $$

### Proof

From Assumption 1, (7), and (10), we have

13 $$\begin{aligned}& \big|\tilde{\beta}_{k}^{\mathrm{BZAU}}\big| \le \biggl\vert \frac{\nabla_{Z}\tilde{f}_{\mu} (z_{k})^{T}(\nabla_{Z}\tilde{f}_{\mu} (z_{k}) - \nabla_{Z}\tilde{f}_{\mu} (z_{k - 1}))}{\eta( - \nabla_{Z}\tilde{f}_{\mu} (z_{k - 1})^{T}d_{k - 1})} \biggr\vert \le\frac{\|\nabla_{Z}\tilde{f}_{\mu} (z_{k})\|L\alpha_{k - 1}\|d_{k - 1}\|}{\eta(a_{1}\alpha_{k - 1}\|d_{k - 1}\|^{2})}, \\& \big|\tilde{\beta}_{k}^{\mathrm{BZAU}}\big|\|d_{k - 1}\| \le \biggl( \frac{L\|\nabla_{Z}\tilde{f}_{\mu} (z_{k})\|}{\eta (a_{1}\|d_{k - 1}\|)}\biggr)\|d_{k - 1}\|=\frac{L\|\nabla_{Z}\tilde{f}_{\mu} (z_{k})\|}{\eta a_{1}}, \end{aligned}$$

i.e.,

$$\big|\tilde{\theta}_{k}^{\mathrm{BZAU}}\big|\|y_{k - 1}\| \le \bigg| \frac{\nabla_{Z}\tilde{f}_{\mu} (z_{k})^{T}d_{k - 1}}{\eta( - \nabla_{Z}\tilde{f}_{\mu} (z_{k - 1})^{T}d_{k - 1})} \bigg|\| y_{k - 1}\|. $$

From Assumption 1, (8), and (10), we have

14 $$ \big|\tilde{\theta}_{k}^{\mathrm{BZAU}}\big|\|y_{k - 1}\| \le \biggl( \frac{\|\nabla_{Z}\tilde{f}_{\mu} (z_{k})\|L\|x_{k} - x_{k - 1}\|}{\eta(a_{1}\alpha_{k - 1}\|d_{k - 1}\|^{2})}\biggr)\|d_{k - 1}\| = \frac{L\|\nabla_{Z}\tilde{f}_{\mu} (z_{k})\|}{\eta a_{1}}. $$

Combining (13), (14), and $d_{k}$ generated in Algorithm 1, we obtain

$$\begin{aligned} \|d_{k}\| &\le\big\| \nabla_{Z}\tilde{f}_{\mu} (z_{k})\big\| + \bigl\vert \tilde{\beta}_{k}^{\mathrm{BZAU}} \big\vert \|d_{k - 1}\| + \bigr\vert \tilde{\theta}_{k}^{\mathrm{BZAU}} \big\vert \|y_{k - 1}\| \\ &\le\big\| \nabla_{Z}\tilde{f}_{\mu} (z_{k})\big\| + \frac{L\|\nabla_{Z}\tilde{f}_{\mu} (z_{k})\|}{\eta a_{1}}+\frac{L\|\nabla_{Z}\tilde{f}_{\mu} (z_{k})\|}{\eta a_{1}} \\ &=\biggl(1 + \frac{2L}{\eta a_{1}}\biggr)\big\| \nabla_{Z} \tilde{f}_{\mu} (z_{k})\big\| .\end{aligned} $$

Denote $\sqrt{B} = (1 + \frac{2L}{\eta a_{1}})$, we have $\|d_{k}\|^{2} \le B\|\nabla_{Z}\tilde{f}_{\mu} (z_{k})\|^{2}$, i.e.,

$$\frac{1}{\|d_{k}\|^{2}} \ge \frac{1}{B\|\nabla_{Z}\tilde{f}_{\mu} (z_{k})\|^{2}} $$

and

$$\frac{B\|\nabla_{Z}\tilde{f}_{\mu} (z_{k})\|^{4}}{\|d_{k}\|^{2}} \ge \frac{\|\nabla_{Z}\tilde{f}_{\mu} (z_{k})\|^{4}}{\|\tilde{g}_{k}\|^{2}} =\big\| \nabla_{Z} \tilde{f}_{\mu} (z_{k})\big\| ^{2} $$

By Lemma 2, we have

$$\sum_{k = 0}^{\infty} \big\| \nabla_{Z} \tilde{f}_{\mu} (z_{k})\big\| ^{2} < + \infty. $$

This completes the proof. □

## Numerical experiments

In this section, we give some numerical experiments of Algorithm 1, which are also considered in [2, 9, 10, 14, 15]. We compare Algorithm 1 with smoothing gradient method, GPSR method, debiased and minimum norm methods proposed in [2, 9, 10, 14] respectively. The numerical results of all the examples show that Algorithm 1 is effective. All codes run in MATLAB 8.0. For Examples 1 and 2, the parameters used in Algorithm 1 are chosen as $\sigma= 0.2$, $\mu= 5$, $\eta= 2$, $\gamma= 0.5$, $\varepsilon= 10^{ - 6}$, $\rho= 0.4$.

### Example 1

Consider

$$A = \left ( \textstyle\begin{array}{c@{\quad}c@{\quad}c@{\quad}c@{\quad}c@{\quad}c} 3 & 5 & 8 & 4 & 1 & 5 \\ 2 & 9 & 6 & 5 & 7 & 4 \\ 3 & 4 & 7 & 2 & 1 & 6 \\ 8 & 9 & 6 & 5 & 7 & 4 \end{array}\displaystyle \right ),\qquad b = ( \textstyle\begin{array}{c@{\quad}c@{\quad}c@{\quad}c} 2 & 4 & 1 & 7 \end{array}\displaystyle )^{T}, $$

and $\tau= 5$.

**Figure 1:**
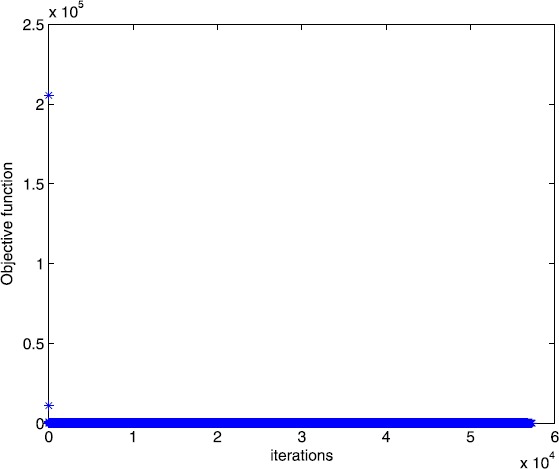
Numerical results for solving Example 1 with Algorithm 1

**Figure 2:**
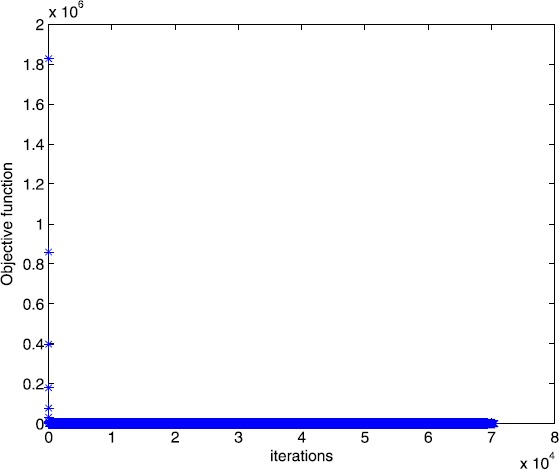
Numerical results for solving Example 1 with smoothing gradient method

From [14], we know that this example has a solution ${x}^{*} = (0.3461,0.0850,0,0,0.3719,0)^{{T}}$. The optimal solution of Algorithm 1 is ${x}^{*} = ( 0.3459, 0.0850,0.0001, 0.0009,0.3717, - 0.0001)^{{T}}$. In Figs. 1 and 2, we plot the evolution of the objective function versus the number of iterations when solving Example 1 with Algorithm 1 and the smoothing gradient method respectively.

### Example 2

**Figure 3:**
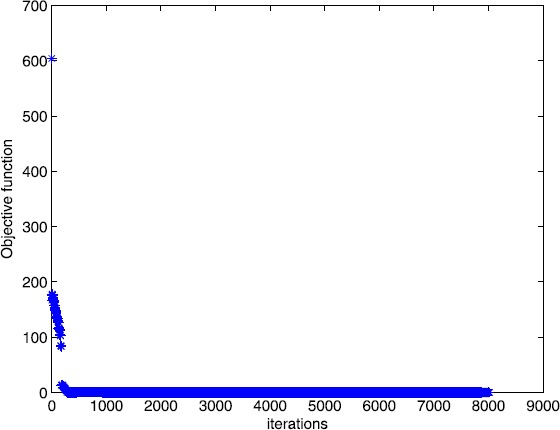
Numerical results for solving Example 2 with Algorithm 1

**Figure 4:**
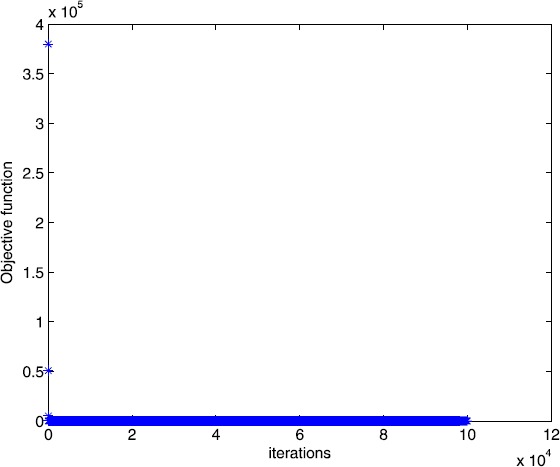
Numerical results for solving Example 2 with smoothing gradient method

Consider

$$\begin{gathered} A = \left ( \textstyle\begin{array}{c@{\quad}c@{\quad}c@{\quad}c@{\quad}c@{\quad}c@{\quad}c@{\quad}c@{\quad}c@{\quad}c@{\quad}c@{\quad}c@{\quad}c} 1 & 1 & 1 & 0 & 0 & 0 & \cdots& 0 & 0 & 0 & 1 & \cdots& 1 \\ 0 & 0 & 0 & 1 & 1 & 1 & \cdots& 0 & 0 & 0 & 1 & \cdots& 1 \\ \vdots& \vdots& \vdots& \vdots& \vdots& \vdots& \cdots& \vdots& \vdots& \vdots& \vdots& \cdots& \vdots\\ 0 & 0 & 0 & 0 & 0 & 0 & \cdots& 1 & 1 & 1 & 1 & \cdots& 1 \end{array}\displaystyle \right )_{m \times n},\\b = ( \textstyle\begin{array}{c@{\quad}c@{\quad}c@{\quad}c} 1 & 1 & \cdots& 1 \end{array}\displaystyle )^{T},\end{gathered} $$

and $\tau= 2$. In this example, we choose $m = 30$, $n = 100$. The numerical results are given in Figs. 3 and 4.

### Example 3

**Figure 5:**
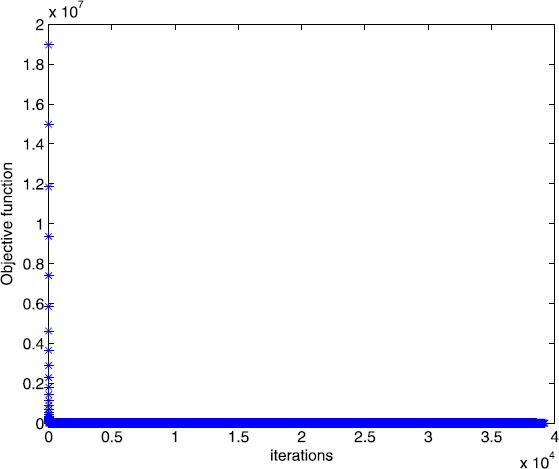
Numerical results for solving Example 3 with Algorithm 1

**Figure 6:**
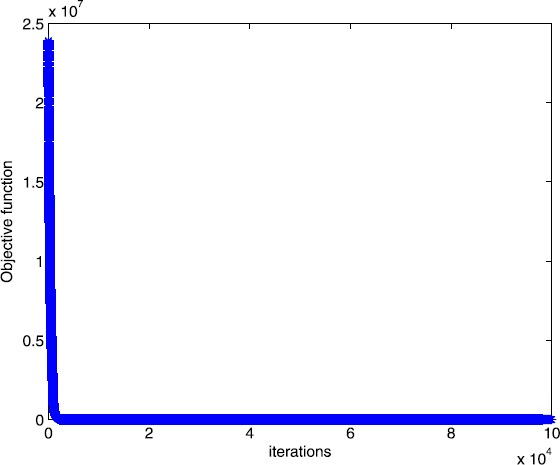
Numerical results for solving Example 3 with smoothing gradient method

Consider

$$A = \left ( \textstyle\begin{array}{c@{\quad}c@{\quad}c@{\quad}c@{\quad}c@{\quad}c@{\quad}c@{\quad}c} 1 & 0 & \cdots& 0 & 1 & 1 & \cdots& 1 \\ 0 & 1 & \cdots& 1 & 0 & 1 & \cdots& 1 \\ \vdots& \vdots& \ddots& \vdots& \vdots& \vdots& \cdots& \vdots\\ 0 & 1 & \cdots& 1 & 0 & 1 & \cdots& 1 \\ 1 & 0 & \cdots& 0 & 1 & 1 & \cdots& 1 \end{array}\displaystyle \right )_{m \times n},\qquad b = ( \textstyle\begin{array}{c@{\quad}c@{\quad}c@{\quad}c} 1 & 1 & \cdots& 1 \end{array}\displaystyle )^{T}, $$

and $\tau= 6$. In this example, we choose $m = 100$, $n = 110$. The numerical results are given in Figs. 5 and 6.

### Example 4

**Figure 7:**
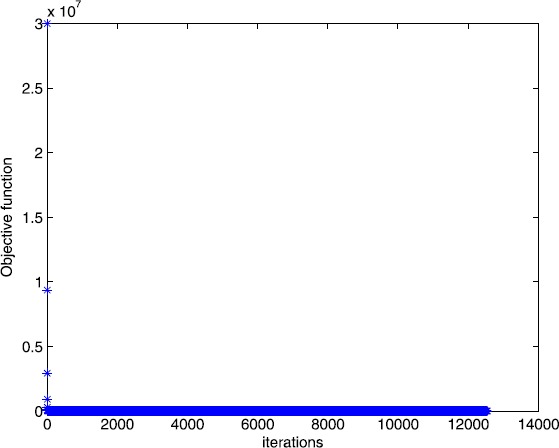
Numerical results for solving Example 4 with Algorithm 1

**Figure 8:**
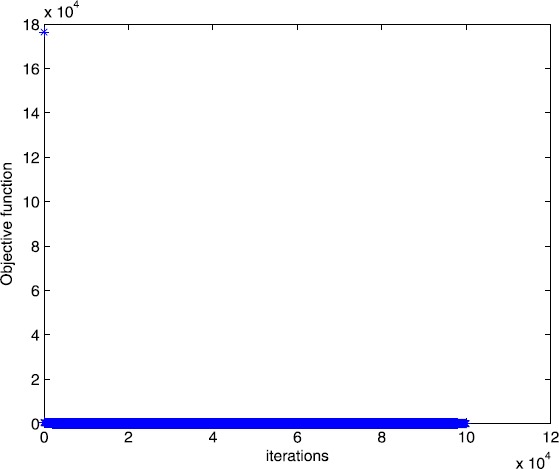
Numerical results for solving Example 4 with smoothing gradient method

Consider

$$A = \left ( \textstyle\begin{array}{c@{\quad}c@{\quad}c@{\quad}c@{\quad}c@{\quad}c@{\quad}c@{\quad}c} 4 & - 1 & 0 & \cdots& 0 & 1 & \cdots& 1 \\ - 1 & 4 & - 1 & \cdots& \vdots& \vdots& \cdots& \vdots\\ 0 & - 1 & 4 & \ddots& \vdots& \vdots& \cdots& \vdots\\ \vdots& \ddots& \ddots& \ddots& - 1 & \vdots& \cdots& \vdots\\ 0 & \cdots& 0 & - 1 & 4 & 1 & \cdots& 1 \end{array}\displaystyle \right )_{m \times n},\qquad b = ( \textstyle\begin{array}{c@{\quad}c@{\quad}c@{\quad}c} 1 & 1 & \cdots& 1 \end{array}\displaystyle )^{T}, $$

and $\tau= 10$. In this example, we choose $m = 200$, $n = 210$. The numerical results are given in Figs. 7 and 8.

### Example 5

**Figure 9:**
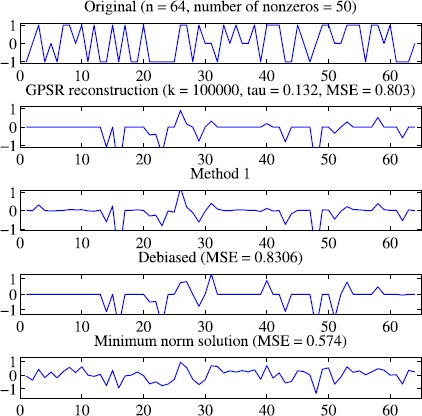
Numerical results for solving Example 5 with Algorithm 1

In this example, we consider a typical compressed sensing problem, which is also considered in [9, 10, 14, 15]. In this example, we choose $m = 2^{4}$, $n = 2^{6}$, $\sigma= 0.5$, $\rho= 0.4$, $\gamma= 0.5$, $\varepsilon= 10^{ - 6}$, $\mu= 5$, $\eta= 2$. The original signal contains 520 randomly generated ±1 spikes. Further, the $m \times n$ matrix *A* is obtained by first filling it with independent samples of a standard Gaussian distribution and then orthogonalization of its rows. In this example, we choose $\sigma^{2} = 10^{ - 4}$ and $\tau= 0.1 \Vert A^{T}y \Vert _{\infty} $ the same as suggested in [14]. The numerical results are shown in Fig. 9.

## Conclusion

In this paper, we have proposed a new smoothing modified three-term conjugate gradient method for solving $l_{1}$-norm nonsmooth problems. Comparing with the smoothing gradient method, GPSR method, and other methods proposed in [2, 9, 10, 14], we can see that the smoothing modified three-term conjugate gradient method is simple and needs small storage. Comparing with the smoothing gradient method proposed in [14], the smoothing modified three-term conjugate gradient method is significantly faster especially in solving large-scale problems.
